# What are patient perspectives on privacy and trust in digital genomic tools? A qualitative study

**DOI:** 10.1002/jgc4.70025

**Published:** 2025-04-30

**Authors:** Vedika Jha, Saumeh Saeedi, Marc Clausen, Daniel Assamad, Sonya Grewal, Daena Hirjikaka, Whiwon Lee, Stephanie Luca, Angela Shaw, Robin Hayeems, Yvonne Bombard, the Genetics Navigator Study Team, Melyssa Aronson, Francois Bernier, Michael Brudno, June C. Carroll, Lauren Chad, Ronald Cohn, Gregory Costain, Irfan Dhalla, Hanna Faghfoury, Jan Friedman, Stacy Hewson, Trevor Jamieson, Rebekah Jobling, Rita Kodida, Anne‐Marie Laberge, Jordan Lerner‐Ellis, Eriskay Liston, Muhammad Mamdani, Christian Marshall, Matthew Osmond, Quynh Pham, Emma Reble, Frank Rudzicz, Emily Seto, Serena Shastri‐Estrada, Cheryl Shuman, Josh Silver, Maureen Smith, Kevin Thorpe, Wendy J. Ungar

**Affiliations:** ^1^ Temerty Faculty of Medicine University of Toronto Toronto Ontario Canada; ^2^ Genomics Health Services Research Program, Li Ka Shing Knowledge Institute, St. Michael's Hospital Unity Health Toronto Toronto Ontario Canada; ^3^ Program in Child Health Evaluative Sciences The Hospital for Sick Children Toronto Ontario Canada; ^4^ Institute of Health Policy, Management and Evaluation University of Toronto Toronto Ontario Canada

**Keywords:** digital health, genetics services, genomics, patient attitudes, privacy

## Abstract

Digital tools have emerged as a promising solution to increase the efficiency and capacity of genomic services. However, accessing information through internet‐based applications raises concerns about privacy and security risks. As patient‐facing digital tools are developed for genomic medicine, it is vital to understand and incorporate patients' perspectives on digital privacy and security. A qualitative study was conducted using semi‐structured interviews and interpretive description. Thirty participants who previously received genetic testing for themselves (*n* = 17) or their child (*n* = 13) were interviewed (*n* = 20 females, *n* = 15 above 50 years old). Participants were willing to store and access genomics personal health information (PHI) in a patient‐facing digital platform. The main benefit identified by participants was the ability to access and control their own PHI. Participants expressed that the benefits of digital genomics services, such as patient empowerment and personalized care, outweighed the perceived risks, such as potential data leaks. In order to minimize risks, participants emphasized the importance of transparency about the security measures in place and who would have access to their PHI. These findings inform the design of digital genomic platforms to enhance patients' sense of security, which is critical for the uptake and usage of any platform.

**What is already known on this topic**

Patient‐facing digital tools are being increasingly used in many medical disciplines, including genetics. However, it is important to ascertain patient perspectives on digital tools and their perceptions of their privacy and security.

**What this study adds**

This study investigates patient perspectives on privacy and security in patient‐facing digital genomics tools, finding that patients are overall willing to store and access genomics personal health information (PHI) in a patient‐facing digital platform. These findings inform the design of digital genomic platforms to enhance patients' sense of security, which is critical for the uptake and usage of any platform.

## 1 INTRODUCTION

Digital health tools are increasingly being used in many medical disciplines to monitor health, improve access to health information, and increase the efficiency of patient–practitioner communication (Bombard et al., 2022; Lee et al., 2023). Within the broader shift toward digital health care, digital genomics tools have emerged as a promising solution to enhance the capacity of genomic services (Bombard & Hayeems, 2020). A digital genomics platform can feature tools including online decision aids to support decisions or software programs, apps, and chatbots to expand genetic counseling, and the delivery of comprehensive genetic services (Bombard et al., 2022). These features can help guide patients throughout their genomics testing journey, including pretest counseling, education, decision‐making, consultation, appointment booking, and return of results (Birch et al., 2016; Bombard et al., 2020; Bombard & Hayeems, 2020; Shickh et al., 2021). Patient‐facing digital genomics tools have been shown to improve patient outcomes such as knowledge, engagement, and well‐being (Lee et al., 2023) and have been well‐received by both healthcare practitioners and patients (Birch et al., 2016; Bombard et al., 2020). They have also been shown to improve efficiency for genetic counselors and other healthcare practitioners, alleviating wait times for genetic services while providing patients with more agency in their care (Bombard et al., 2022; Lee et al., 2023). However, digital health tools come with substantial changes to the storage and security of personal health information (PHI), that is, any information that can be used to identify an individual, including personal and family health history, past medical care received, and healthcare numbers (Personal Health Information Protection Act, 2004, S.O. 2004, c. 3, Sched. A, 2004).

Both patients and practitioners have expressed concerns regarding the digitization of genomics care and the new security risks that it poses (Andrews et al., 2020; Arshad et al., 2021; Bonomi et al., 2020; Heeney et al., 2011; O'Connor et al., 2016; Rogith et al., 2014). These concerns may be heightened due to the sensitive nature of genomic information that, if leaked or hacked, could affect not only the patients but the patients' relatives (Bonomi et al., 2020; O'Connor et al., 2016). Healthcare breaches are becoming more frequent globally (Seh et al., 2020). In the United States, 133 million health records were breached in 2023, and in Canada, attacks are also increasing, with nearly 13 million records breached in 2019 (Harish et al., 2023). The majority of healthcare breaches are due to hacking (Seh et al., 2020; Vayena et al., 2018).

Digital genomics tools contain different kinds of sensitive health information with varying degrees of anonymity. For example, digital tools used by labs and sequencing facilities may contain information about a patient's DNA sequence and their health record number but minimal personal identifiers (Bombard et al., 2022). On the other hand, a patient‐facing tool may not contain DNA sequence information but may contain PHI in the form of personal health history, family history, health record number, or results from other genetic tests (Birch et al., 2016; Bombard et al., 2020, 2022), which can identify the patient. Thus, a significant issue for digital genomics platforms is the privacy and security of information. The success of any digital health tool is dependent on patients trusting the platform in order to consent to having their genetic test results and other PHI uploaded to and maintained on the platform. A 2016 review exploring the factors that affect engagement with and recruitment to digital health interventions identified privacy and security concerns as a key deterrent to using digital tools (O'Connor et al., 2016).

Ethical frameworks concerning digital health tools have explored the risk‐benefits involved, focusing on the importance of shared decision‐making and balancing privacy with accessibility (Rahimzadeh et al., 2020; Ruotsalainen & Blobel, 2020). Commentators have asserted that individuals must be able to maintain some rights over personal data along with the data custodians, but once patients are given access to their own genomics data, they too share the responsibility for maintaining confidentiality (Karabekmez et al., 2021; Rahimzadeh et al., 2020). This is particularly relevant to genomics data, as a confidentiality breach could impact not only the patient but also their family members (Karabekmez, 2021; Rahimzadeh et al., 2020; Ruotsalainen & Blobel, 2020) Privacy and trust are also interconnected; trust must be fostered between the data custodians and the patients to ensure transparency and optimize engagement. Current guidelines on digital genomics are broad and largely revolve around data sharing between researchers rather than patient‐facing digital tools. However, many similar and relevant themes emerge. The American College of Medical Genetics (ACMG)'s guidelines emphasize patient engagement and education and the Global Alliance for Genomics and Health (GA4GH) Accountability Policy shared responsibility for data stewardship (Knoppers & Thorogood, 2017). While not specific to digital genomics, Canada's 2017 Genetics Nondiscrimination Act protects individual's privacy by ensuring that insurers, employers, and others cannot request, use, or disclose genetic test results without written consent (Cowan et al., 2022).

In genomics, most of the current literature on the privacy and security of digital tools focuses on the concerns of healthcare practitioners and recommendations on how to improve privacy and security, such as establishing regulatory frameworks and guidance on how to mitigate privacy risks through methods like encryption and secure backup (Arshad et al., 2021; Bonomi et al., 2020; Heeney et al., 2011). Existing literature on patient perspectives explores genomic data sharing for research purposes (Andrews et al., 2020; Rogith et al., 2014). There is a paucity of evidence describing patient perspectives on the privacy and security of digital genomics tools. In this study, we aimed to assess patient perspectives on privacy and trust in digital genomic tools, a critical step to inform the design of digital genomic platforms and facilitate patients' trust in such systems (Bonomi et al., 2020).

## 2 METHODS

### 2.1 Design

We conducted a qualitative study using semi‐structured interviews to assess the preferences of adult patients and parents of pediatric patients regarding privacy, security, and trust related to the use of digital genomics tools. We employed interpretive description in order to capture key ideas from the interviews and a variety of perspectives from research participants (Hunt, 2009). Research ethics approval was obtained through St. Michael's Hospital, Toronto, Canada. Consent was obtained for study participation and audio recording.

### 2.2 Sample and recruitment

Inclusion criteria included English proficiency (because interviews were conducted in English) and previous experience with any type of genetic testing for any type of presenting condition for the participant or their child of any age. Patients and parents of patients who had undergone genetic testing related to a presenting genetic condition were interviewed to capture the perspectives of people who are familiar with the existing system of genetic testing and who represent potential users of a digital genomics tool. Herein, the term “patient perspectives” is used to describe the perspectives of both patients themselves and their caregivers. Potential participants were recruited either through an invitation and reminder email sent by the Canadian Organization of Rare Disorders (CORD) or were previous participants in a Toronto‐based cancer genetics study and had agreed to be recontacted for future research. We aimed to sample nationally to capture a diverse range of perspectives and experiences with different provincial healthcare systems. Participants were offered a $20 gift card in appreciation of their time. Those who were interested in the study contacted the study team and were screened to ensure they met the eligibility criteria. Recruitment occurred between January and July of 2021.

### 2.3 Data collection

We conducted semi‐structured interviews over the telephone or using video conferencing software. Interviewees were aware that this interview was part of a needs assessment in advance of developing a digital genomics tool designed for parents of minor patients and adult patients. Additionally, participants completed a short demographics survey asking about gender, age, education level, and region of residence. During the interview, participants were asked a range of questions related to the privacy, security, and credibility of digital platforms. Questions included “What would make a digital genomics platform feel secure?” and “What would make a digital genomics platform credible?” The interview guide is provided in Appendix 3. Specifically, participants were asked about storing their personal health information and family health history on a digital platform and receiving results of genetic testing through a digital platform. Participants were not asked about their perspectives on storing their DNA sequences on a digital genetics platform.

Participants were then asked to share what features would make them trust the information on the platform and what features would allow them to trust the platform to hold their personal health information. Since this study was hypothetical and did not pertain to a specific digital genomics tool, personal health information was not explicitly defined to participants.

Four researchers who all had Master's level degrees, previous interview experience, and an interest in improving genetic services conducted the interviews (AS: research assistant, WL: genetic counselor, SL: research project manager, and MC: research program manager). All but one of the interviewers was female. No one else was present for the interviews.

All interviewers were previously unknown to the interviewee. For each researcher, the first two interviews were conducted with another member of the research team present to maximize consistency. Informed by the first few interviews, the interview guide was revised to improve clarity and build upon emerging themes. After the interviews, participants were offered an opportunity to review their transcripts if requested and provide additional feedback. The average interview was 53 min 16 s. Interviews occurred between January and July of 2021. Interviews were completed once it was agreed that data saturation had been achieved. No repeat interviews were conducted.

### 2.4 Analysis

Interviews were digitally recorded and transcribed verbatim for analysis. Interpretive description employing thematic analysis was used to analyze interview transcripts (Braun & Clarke, 2022). The primary researcher (VJ) developed the coding scheme by reading the interview transcripts and consulting with the study team about key themes in the data. The coding structure was fine‐tuned and reviewed by a second researcher (SS). The coding scheme is provided in Appendix 4. Both researchers then independently coded the first four interviews using Dedoose software (Version 9.0.54, Los Angeles) (SocioCultural Research Consultants, LLC, 2021) and compared their coding in order to ensure mutual understanding of the codes. After discussion between both researchers and the study team about preliminary themes and patterns, the remaining coding was completed by the primary researcher and reviewed by the second researcher to ensure adherence to the agreed‐upon coding scheme. The researchers met throughout the process to discuss impressions, and disagreements were resolved through discussion between the two researchers and consultation with the study team. Participants did not provide feedback. Researchers also discussed reflexivity to address potential biases and assumptions they may have. Final themes stemming from the coded transcripts were presented and discussed with the study team to develop an overall understanding of patient perspectives and draw conclusions. Data analysis occurred between May and August 2022.

## 3 RESULTS

### 3.1 Demographic characteristics

Participant demographics were previously reported by Luca et al. (2023) who studied the same population focusing on a different topic from the interview guide (Luca et al., 2023). Thirty participants who previously received genetic testing for themselves (*n* = 17) or their child (*n* = 13) were interviewed. There were no substantive differences between the perspectives of patients or parents of patients. No participants dropped out of the study. Participants were all at least 30 years of age, and 50% were over 50 years of age. Twenty participants identified as female and 10 identified as male. Twenty‐seven participants (90%) had a university or college degree or higher education. Over one‐third of participants (*n* = 22) lived in an urban center (Table 1).

**TABLE 1:** Participant demographics, adapted from Luca et al. (2023).

Participant type	
Patient	17
Parents of patients	13^a^
Age	
30–49 years	15
50+ years	15
Gender identity	
Male	10
Female	20
Education level	
High school graduate	3
College/university graduate	13
Graduate degree or greater	14
Primary language spoken at home^b^	
English	26
French	4
Other	2
Region in Canada	
Québec	3
Ontario	21
West & Prairie	6
Other	2
Population size of residence area	
Large urban population (100,000 or more people)	22
Medium population center (30,000–99,999 people)	4
Small or rural population (Less than 30,000 people)	4
Family history of genetic disease?	
Yes	12
No	17
Unknown	1

^a^ Parents included seven mothers and six fathers, all participants, but one was one of the child's primary caregivers.

^b^ Two participants chose more than one option.

### 3.2 Patient perspectives on privacy and trust in digital genomic tools

Overall, participants were willing to access and store PHI through a digital genomics platform but acknowledged that doing so comes with risks. On balance, participants indicated that aspects of digital genomics services, such as tailoring digital tools to patient needs and increasing patients' access to and agency in their care, were perceived to be beneficial and outweighed the perceived risks, such as potential data leaks. Three main themes emerged from the analysis to summarize patient perspectives: risk tolerance, minimizing risks, and maximizing benefits. Each theme is developed below.

### 3.3 Risk tolerance

When asked about privacy, security, and trust, many participants indicated low levels of concern and a high level of risk tolerance. Many participants could not identify any serious risks of accessing and storing their genetic results on a digital platform. Those who expressed concern worried about insurance companies accessing their PHI or that their personal data could be sold to third parties and used for commercial purposes.

To be honest, like at the end of the day, like I'm not too worried about the data, like it's there, you know like if somebody else, like if there was a breach of that and, you know like I just don't know how it could be used nefariously…I'm not too worried about that piece, to be honest, cause it's a tool that we use to help care for our son. (PAR007)

Participants described a broader shift in how personal information is stored and accessed today. Multiple participants highlighted how an increasing amount of sensitive information, such as banking information, is stored digitally with similar user‐facing platforms that allow electronic access to personal information and referenced the high degree of trust required to use digital banking services. In comparison, most participants expressed that storing genetic PHI digitally seems far less risky than storing financial information.

You know, it's very funny how we take all our life savings and we put it into virtual platforms, in essence, but we really worry about some of our medical information, which I feel like is far less risky, obviously if it was disclosed. (PAR010)

Some participants discussed that they are overall more tolerant of risk due to a general sentiment that, as more and more traditionally in‐person services shift online, it is inevitable for genetic services to shift to online platforms as well. This feeling of inevitability among participants decreased some concerns surrounding safety measures.

Eventually everything's gonna be online. Eventually everything's gonna be done through electronic media. (PAR003)

However, the feeling of inevitability manifested differently for some, who doubted that anything kept online could ever be truly secure and felt there would always be a risk of data breach.

But really, anything that's really secure, is really, not really, you know, deep down in the rabbit hole, I mean, everyone has access to everything. (PAT007)

Further discussions highlighted ways to minimize the risks associated with a digital genomics platform and ways to maximize its benefits.

### 3.4 Minimizing risks

Despite the generally high level of risk tolerance for using a digital genomics platform, participants still expected to have basic security measures in place, such as a username and password. Some participants took this expectation a step further, noting that the platform should be encrypted so that patient information would be incomprehensible to nonauthorized parties. Other participants wanted to ensure that all data are stored in Canada. However, within these discussions, many participants admitted to not understanding aspects of security and how information is protected, and thus were unsure about what security measures are adequate.

So long as they're saying they're a secure website by whatever means they're doing it, I mean, that's good enough for me as a layperson. (PAT007)

Partly due to the lack of understanding of the technical details of security, participants voiced that they wanted the privacy and security measures to be clearly detailed in plain language somewhere on the platform. Participants wanted a clear explanation of who has access to the data, where the data are being kept, how the data are being secured, and how they could remove the data from the platform should they choose to do so. Some expressed that this should be captured in a consent form, while others wanted to have an information page on the platform. Either way, participants clearly valued having as much transparency and clarity as possible about a platform's privacy and security measures.

There are certain sorts of things that make me trust bank sites, and, any other, digital site, where there is, there's obvious indicators and information available about the levels and the types of encryption that are used. (PAR004)

I think having that information made really clear would be helpful and comforting to most people. And not in just some tiny little writing, but really upfront. (PAT008)

When asked what would make them trust a platform's credibility, most participants discussed measures such as including institutional logos of a hospital or university with which the platform is associated. An institutional logo that they already trust with their healthcare and PHI increased their comfort in storing their PHI on a platform. Participants also mentioned other indicators of credibility such as citations from peer‐reviewed journals for any general health information on the platform. Participants remarked how these kinds of indicators of credibility do not mean that the information is actually secure, but since participants already view these institutions as reputable and trustworthy, they assumed that adequate security measures would be put in place.

If it comes from the hospital, I'm gonna assume the hospital knows what they're doing. Like I see the same report that the doctor sees online. It makes people secure and happy with that. Trust, so, that's the trust being met, yes, definitely. (PAR003)

### 3.5 Maximizing benefits

Some participants described how accessing PHI online may come with risks, especially in the realm of genetics. However, participants brought up potential benefits of a digital genomics platform that would outweigh risks, such as the ability to readily access their own information and have more agency and control over their health care. Many participants want access to their full test results and want to get as much information as possible, which they feel could be facilitated through digital platforms. Digital tools can be tailored to fit patient preferences and allow patients to access and store information in a way most suited to their needs. Digital storage of PHI allows for easier and quicker sharing of health results between different healthcare practitioners, which is particularly useful for patients with complex genetic conditions who have had multiple rounds of genomic tests or other health assessments. It also means that patients could have the ability to easily share their information with future healthcare practitioners, making their care more efficient.

As long as it's just the people that you have said could have access to your data, have, you know, are the ones that are doing that. I don't, certainly don't have huge issues with that. If that data is going to optimize your care, that's great, that's wonderful! (PAR001)

You can have a digital videos and programs, and something that you log into…to the best of your knowledge and expertise, [you] have read up and have a medium to good understanding of the problem, and then, can consult comfortably with the healthcare professionals. (PAR005)

Increased access and control over personal data was viewed not only as a benefit of digital care but also as a benefit for security. Participants felt as though their data would be most secure if it was clear that the patient had control over keeping and sharing data within the platform and would be able to remove their personal information from the platform at any time. However, it is important to note that patients removing information from a digital platform would not modify their medical chart and would only remove information from the shared digital platform.

…A few points that shows me, I'm in control. Like you are getting my information because I allow it. OK, and I can remove that, I can revoke that back at any time, OK. So something that shows me that the element of control is really good. (PAT005)

Overall, participants highlighted the importance of agency, control, and efficiency. Participants indicated that these three values would make them feel more secure using a digital platform as well as provide them with the most benefit out of the platform.

## 4 DISCUSSION

As the use and storage of PHI in digital health tools in genetic services increase, it is imperative to consider patient perspectives on the privacy and security of their own information. This qualitative study provides timely insights into patient attitudes toward the privacy and security of patient‐facing digital genomics platforms. Participants were overall willing to access their PHI through a digital platform and expressed that the benefit of personalizing their care outweighed potential risks such as data leaks. The broader societal shift to digital information storage contributed to participants' willingness to store PHI on digital platforms. To minimize risks, participants felt that the security measures of any digital genomics platform should be clearly explained in plain language to ensure that users understand how their information is being protected. Participants recommended the inclusion of several indicators of credibility, such as institutional logos or citations of peer‐reviewed articles that can bolster their trust in using and storing PHI on a digital platform. Last, participants emphasized the importance of patient access and control over their own PHI and saw this enhanced level of agency and efficiency as the main benefit of a patient‐facing platform. Participants indicated that these perceived benefits exceeded risks regarding privacy and security concerns.

Our work contributes to the existing literature on privacy in digital health tools, adding the perspective of patients on patient‐facing digital genomic tools. This perspective has not previously been explored in other studies that have asked about digitizing genomic information for research purposes or have looked at patient‐facing health tools not specific to genomics, such as cancer treatment or general medical history storage. Similar to our results, those studies on data sharing or patient‐facing tools found a high level of risk tolerance among participants and low levels of concern about the privacy of (research) genomic or general health data compared to banking or social security information (Rogith et al., 2014; Spencer et al., 2016). Further, participants who had privacy concerns about patient‐facing health tools still supported digital tool development or chose to participate in the digital intervention of the study, indicating that the benefits outweighed risks (Wetzels et al., 2018; Abdulhussein et al. 2023; Busch‐Casler et al., 2023; Chow et al. 2024). When asked about privacy concerns with digital PHI, participants identified risks related to their information being leaked or sold to insurance companies in both patient‐facing and research contexts (Andrews et al., 2020; Gupta et al., 2023; Papoutsi et al., 2015; Rogith et al., 2014; Spencer et al., 2016; Vodicka et al., 2015; Wetzels et al., 2018). Another study also found feelings of inevitability and resignation among participants (Gupta et al., 2023). Other studies have also shown patient preference for institutional logos in digital health tools, emphasizing the importance of oversight and maintenance of tools by established healthcare institutions to support a platform's credibility (Busch‐Casler et al., 2023; Walters et al., 2023). Results from several studies emphasized transparency and patient control over data sharing (Gupta et al. 2023; Spencer et al., 2016; Vodicka et al., 2015; Wetzels et al., 2018) and found that participants valued increased knowledge and efficiency in care (Papoutsi et al., 2015). These similarities show that even across multiple contexts and health conditions, patients value transparency, access to, and control of information in digital genomic tools. Our findings extend these trends by providing the perspective of patients on patient‐facing genomic digital tools.

There are limitations to this study. First, participants were not asked about the kind of genetic testing that they or their children previously underwent, their presenting condition, or whether they had a positive, uncertain, or negative result. Perspectives may vary across different disease populations as well as across participants with different genetic testing experiences. Additionally, this study was hypothetical in nature and did not ask patients about a specific genomics digital tool, leaving room for participants to interpret what the tool would entail and how it would fit into patient care. However, participants were told that, hypothetically, the PHI presented through the tool would include personal identifiers, past medical history, and family health history as well as genetic test results, but would not include DNA sequence information. This specification helped direct participants' interpretation of the proposed digital genomic tool and allowed them to provide broad perspectives on what this tool should include. Participants could use their existing experience with other digital health tools to shape these perspectives. Two‐thirds of our participants identified as female, which may skew the results if they differ by gender, but no prior literature has suggested gender differences. Despite efforts to recruit patients living in rural or remote communities, most of our participants resided in urban settings and were highly educated, limiting the transferability of results. We did not record the ethnicity of participants either, and results may differ across different cultural backgrounds.

Future studies could expand upon our work to capture a more diverse array of patient perspectives, such as a greater variety of geographic settings, ethnicities, and education levels. Specific disease populations can also be investigated in order to understand their specific needs in digital tools. Doing so would help tailor digital genomics tools to different patient groups, building the capacity of digital genomics care in a variety of clinical settings. Additionally, further research could investigate patient perspectives after using a specific genomics digital tool, allowing participants to reflect on their experiences and provide more insight into how genomics digital tools can be improved. This will help create patient‐friendly platforms that meet the needs of patients, hence increasing their usage. Future platforms designed with patient perspectives in mind can enhance genetic counseling care delivery by decreasing wait times without compromising patient‐centered care.

This qualitative study provides novel insights into patient and parental attitudes toward privacy and trust in patient‐facing digital genetics platforms. This study identified a high tolerance for risk among participants and highlighted key themes of agency, control, and efficiency. These themes can inform the development of core principles that should be prioritized when developing patient‐facing genetics platforms and may take the form of clear explanations, credibility indicators, and patient control of information sharing. Doing so will enhance patients' sense of security, which is critical for the uptake and usage of any platform.

## AUTHOR CONTRIBUTIONS

Conceptualization: Vedika Jha, Marc Clausen, Robin Hayeems and Yvonne Bombard. Data curation: Vedika Jha, Saumeh Saeedi, Stephanie Luca, Marc Clausen, Daniel Assamad, Sonya Grewal and Daena Hirjikaka. Formal analysis: Vedika Jha, Saumeh Saeedi, Daniel Assamad, Sonya Grewal, Marc Clausen, Stephanie Luca, Yvonne Bombard and Robin Hayeems. Funding acquisition: Yvonne Bombard and Robin Hayeems. Investigation: Vedika Jha, Daniel Assamad, Saumeh Saeedi, Sonya Grewal and Marc Clausen. Methodology: Marc Clausen, Angela Shaw, Robin Hayeems and Yvonne Bombard. Project administration: Vedika Jha, Stephanie Luca, Marc Clausen, Saumeh Saeedi, Daniel Assamad, Sonya Grewal, Daena Hirjikaka, Whiwon Lee and Angela Shaw. Resources: Yvonne Bombard and Robin Hayeems. Software: Vedika Jha, Marc Clausen, Daniel Assamad, Sonya Grewal, Saumeh Saeedi and Daena Hirjikaka. Supervision: Yvonne Bombard and Robin Hayeems. Validation: Vedika Jha, Saumeh Saeedi, Marc Clausen, Daniel Assamad, Sonya Grewal, Daena Hirjikaka, Whiwon Lee, Stephanie Luca, Angela Shaw, Robin Hayeems and Yvonne Bombard. Visualization: Vedika Jha, Saumeh Saeedi, Marc Clausen and Stephanie Luca. Writing – original draft: Vedika Jha, Saumeh Saeedi and Stephanie Luca. Writing – review and editing: Vedika Jha, Saumeh Saeedi, Marc Clausen, Daniel Assamad, Sonya Grewal, Daena Hirjikaka, Whiwon Lee, Stephanie Luca, Angela Shaw, Robin Hayeems and Yvonne Bombard.

## FUNDING INFORMATION

This research was funded by the McLaughlin Centre, University of Toronto, and the Canadian Institutes of Health Research Project Grant—Bridge Funding (Funding Reference Numbers: PMJ 175409 and PNN 177934).

## CONFLICT OF INTEREST STATEMENT

The authors have no relevant financial or nonfinancial interests to disclose.

## ETHICS STATEMENT

Human studies and informed consent: Research ethics approval was obtained through the Unity Health Toronto Research Ethics Board (REB# 20‐143). Informed verbal consent was provided by all participants prior to inclusion in the study. Animal studies: No animal studies were involved in this project.

## Data Availability

Redacted transcripts are available upon request.

## APPENDIX 1

All Genetics Navigator Study Team members are to be indexed in PubMed: Melyssa Aronson (Temerty Faculty of Medicine, University of Toronto, Toronto, ON, Canada; Zane Cohen Centre for Digestive Diseases, Sinai Health System, Toronto, ON, Canada), Francois Bernier (Department of Medical Genetics, Alberta Children's Hospital, Calgary, AB, Canada), Michael Brudno (HPC4Health Consortium, Toronto, ON, Canada), June C. Carroll (Department of Family & Community Medicine, Sinai Health, University of Toronto, Toronto, ON, Canada), Lauren Chad (Division of Clinical and Metabolic Genetics, The Hospital for Sick Children, Toronto, ON, Canada; Department of Pediatrics, The Hospital for Sick Children, Toronto, ON, Canada; Department of Bioethics, The Hospital for Sick Children, Toronto, ON, Canada), Ronald Cohn (Division of Clinical and Metabolic Genetics, The Hospital for Sick Children, Toronto, ON, Canada; Program in Genetics & Genome Biology, The Hospital for Sick Children, Toronto, ON, Canada), Gregory Costain (Temerty Faculty of Medicine, University of Toronto, Toronto, ON, Canada; Division of Clinical and Metabolic Genetics, The Hospital for Sick Children, Toronto, ON, Canada), Irfan Dhalla (Institute of Health Policy, Management and Evaluation, University of Toronto, Toronto, ON, Canada; Care Experience Institute, Unity Health Toronto, Toronto, ON, Canada), Hanna Faghfoury (Fred A. Litwin Family Centre in Genetic Medicine, University Health Network and Sinai Health System, Toronto, ON, Canada), Jan Friedman (Department of Medical Genetics, University of British Columbia, Vancouver, BC, Canada), Stacy Hewson (Temerty Faculty of Medicine, University of Toronto, Toronto, ON, Canada; Division of Clinical and Metabolic Genetics, The Hospital for Sick Children, Toronto, ON, Canada), Trevor Jamieson (Department of Medicine, University of Toronto, Toronto, ON, Canada), Rebekah Jobling (Division of Clinical and Metabolic Genetics, The Hospital for Sick Children, Toronto, ON, Canada), Rita Kodida (Genomics Health Services Research Program, Li Ka Shing Knowledge Institute, St. Michael's Hospital, Unity Health Toronto, Toronto, ON, Canada), Anne‐Marie Laberge (Division of Medical Genetics, Department of Pediatrics, Centre Hospitalier Universitaire Sainte‐Justine and Université de Montréal, Montreal, QC, Canada), Jordan Lerner‐Ellis (Lunenfeld‐Tanenbaum Research Institute, Sinai Health System, Toronto, ON, Canada; Department of Laboratory Medicine and Pathobiology, University of Toronto, Toronto, ON, Canada), Eriskay Liston (Temerty Faculty of Medicine, University of Toronto, Toronto, ON, Canada; Division of Clinical and Metabolic Genetics, The Hospital for Sick Children, Toronto, ON, Canada), Muhammad Mamdani (Genome Diagnostics, Department of Pediatric Laboratory Medicine, The Hospital for Sick Children, Toronto, ON, Canada), Christian Marshall (Department of Laboratory Medicine and Pathobiology, University of Toronto, Toronto, ON, Canada; Genome Diagnostics, Department of Pediatric Laboratory Medicine, The Hospital for Sick Children, Toronto, ON, Canada), Matthew Osmond (Children's Hospital of Eastern Ontario Research Institute, University of Ottawa, Ottawa, ON, Canada), Quynh Pham (Institute of Health Policy, Management and Evaluation, University of Toronto, Toronto, ON, Canada; Centre for Digital Therapeutics, University Health Network, Toronto, ON, Canada), Emma Reble (Genomics Health Services Research Program, Li Ka Shing Knowledge Institute, St. Michael's Hospital, Unity Health Toronto, Toronto, ON, Canada), Frank Rudzicz (International Centre for Surgical Safety, Li Ka Shing Knowledge Institute, St. Michael's Hospital, Unity Health Toronto, Toronto, ON, Canada), Emily Seto (Institute of Health Policy, Management and Evaluation, University of Toronto, Toronto, ON, Canada; Centre for Digital Therapeutics, University Health Network, Toronto, ON, Canada), Serena Shastri‐Estrada (Genetics Navigator Advisory Board, Toronto, ON, Canada; Department of Occupational Science and Occupational Therapy, Temerty Faculty of Medicine, University of Toronto, Toronto, ON, Canada), Cheryl Shuman (Temerty Faculty of Medicine, University of Toronto, Toronto, ON, Canada), Josh Silver (Temerty Faculty of Medicine, University of Toronto, Toronto, ON, Canada; Fred A. Litwin Family Centre in Genetic Medicine, University Health Network and Sinai Health System, Toronto, ON, Canada), Maureen Smith (Patient Partner, Canadian Organization for Rare Disorders, Toronto, ON, Canada), Kevin Thorpe (Dalla Lana School of Public Health, University of Toronto, Toronto, ON, Canada), Wendy J. Ungar (Program in Child Health Evaluative Sciences, The Hospital for Sick Children, Toronto, ON, Canada; Institute of Health Policy, Management and Evaluation, University of Toronto, Toronto, ON, Canada).

## APPENDIX 2 COREQ CHECKLIST

**Table:**

No.	Item	Guide questions/description
**Domain 1: Research team and reflexivity**		
Personal characteristics		
1	Interviewer/facilitator	Which author/s conducted the interview or focus group? Methods Data collection paragraph 3 (page 10)
2	Credentials	What were the researcher's credentials? For example, PhD, MD Methods Data collection paragraph 3 (page 10)
3	Occupation	What was their occupation at the time of the study? Methods Data collection paragraph 3 (page 10)
4	Gender	Was the researcher male or female? Methods Data collection paragraph 3 (page 10)
5	Experience and training	What experience or training did the researcher have? Methods Data collection paragraph 3 (page 10)
Relationship with participants		
6	Relationship established	Was a relationship established prior to study commencement? Methods Data collection paragraph 4 (page 10)
7	Participant knowledge of the interviewer	What did the participants know about the researcher? For example, *personal goals, reasons for doing the research* Methods Data collection paragraph 1 (page 9)
8	Interviewer characteristics	What characteristics were reported about the interviewer/facilitator? For example, *Bias, assumptions, reasons and interests in the research topic* Methods Data collection paragraph 3 (page 10)
**Domain 2: study design**		
Theoretical framework		
9	Methodological Orientation and Theory	What methodological orientation was stated to underpin the study? For example, *grounded theory, discourse analysis, ethnography, phenomenology, content analysis* Methods Design paragraph 1 (page 8)
Participant selection		
10	Sampling	How were participants selected? For example, *purposive, convenience, consecutive, snowball* Methods Sample and recruitment paragraph 1 (pages 8–9)
11	Method of approach	How were participants approached? For example, *face‐to‐face*, *telephone*, *mail*, *email* Methods Sample and recruitment paragraph 1 (pages 8–9)
12	Sample size	How many participants were in the study? Results demographic characteristics paragraph 1 (page 11)
13	Nonparticipation	How many people refused to participate or dropped out? Reasons? Results demographic characteristics paragraph 1 (page 11)
Setting		
14	Setting of data collection	Where was the data collected? For example, *home, clinic, workplace* Methods Data collection paragraph 1 (page 9)
15	Presence of nonparticipants	Was anyone else present besides the participants and researchers? Methods Data collection paragraph 3 (page 10)
16	Description of sample	What are the important characteristics of the sample? For example, *demographic data, date* Results Demographic characteristics paragraph 1 (page 11)
Data collection		
17	Interview guide	Were questions, prompts, and guides provided by the authors? Was it pilot‐tested? Results Demographic characteristics paragraph 1 (page 11)
18	Repeat interviews	Were repeat interviews carried out? If yes, how many? Methods Data Collection paragraph 4 (page 10)
19	Audio/visual recording	Did the research use audio or visual recording to collect the data? Methods Analysis paragraph 1 (page 10–11)
20	Field notes	Were field notes made during and/or after the interview or focus group? Methods Analysis paragraph 1 (page 10–11)
21	Duration	What was the duration of the interviews or focus group? Methods Data collection paragraph 4 (page 10)
22	Data saturation	Was data saturation discussed? Methods Data collection paragraph 4 (page 10)
23	Transcripts returned	Were transcripts returned to participants for comment and/or correction? Methods Data collection paragraph 4 (page 10)
**Domain 3: analysis and findings**		
Data analysis		
24	Number of data coders	How many data coders coded the data? Methods Analysis paragraph 1 (page 10–11)
25	Description of the coding tree	Did the authors provide a description of the coding tree? Methods Analysis paragraph 1 (page 10–11)
26	Derivation of themes	Were themes identified in advance or derived from the data? Methods Analysis paragraph 1 (page 10–11)
27	Software	What software, if applicable, was used to manage the data? Methods Analysis paragraph 1 (page 10–11)
28	Participant checking	Did participants provide feedback on the findings? Methods Analysis paragraph 1 (page 10–11)
Reporting		
29	Quotations presented	Were participant quotations presented to illustrate the themes/findings? Was each quotation identified? For example, *participant number* Yes (results section, pages 11–17)
30	Data and findings consistent	Was there consistency between the data presented and the findings? Yes (results and discussion)
31	Clarity of major themes	Were major themes clearly presented in the findings? Yes (results headings, pages 11–17)
32	Clarity of minor themes	Is there a description of diverse cases or discussion of minor themes? Yes (results and discussion)

## APPENDIX 3 INTERVIEW GUIDE

Thank you so much for taking the time to participate in this study. During this interview, I will be asking you about your experience with virtual care and asking your thoughts on the appropriateness and delivery of virtual care for genetics. A reminder that your responses are confidential. Do you have any questions before we begin?

### 1 Experience with virtual care

With the COVID‐19 pandemic, a lot of us have had exposure to virtual medical care for the first time. What are your thoughts on the shift to digital care these days? Do you have any experience with virtual care in genetics?

By virtual care, I mean communicating with or receiving medical information from your care team online.

Some examples include:

- video calls with your doctor
- online educational modules
- web‐based portals
- chatbots
- digital symptom trackers
- online access to your medical chart (e.g. myChart)
- checking blood work and other results online.

For the purposes of this interview, we are not including phone conversations as virtual care.

If there is no virtual genetics care experience, ask about other types of medical care provided digitally.

- Describe virtual genetics care experience.
- What about virtual care did you like? What did not you like?
- What elements work well virtually and what elements are better suited in person? What is the trade‐off for each approach? What do you gain or lose by in‐person versus digital? What are the risks and benefits?

If a participant has no experience with virtual genetics care, first ask them to describe their virtual care experience for other types of medical care. Then, ask them to imagine the digital experience in genetics, stepwise.

For participants with genetics virtual care experience, map the conversation to the genetic testing journey. For steps they do not have experience with digitally, ask them to imagine digital care for these steps.

Steps are:

- Assessment (family history, medical history, phenotyping)
  ⚬ Examples: filling out family history/symptoms online vs. in person, use of photography
- Education & Counseling (test options, risk/benefits, education, values and decision needs, and personalized counseling support)
  ⚬ Building a relationship with a provider, receiving information, and making decisions based on what is presented to you online vs. an in‐person conversation.
- Return of results and counseling (personalized results explanation, determine support needs, initiate support, and management)
  ⚬ Focus the conversation here. This is where you really want to probe. Probe about comfort level with receiving results online. This includes the result of the test itself as well as additional information that helps to explain what it means. What are the benefits? What are the risks? What would you prefer? Why? How do views change when they think about positive, negative, and uncertain results as well as secondary variants and pharmacogenomic variants? Emotional component
  ⚬ What do they think about being recontacted for re‐analysis of data and updated results?

What about a hybrid model (i.e., a combination of face‐to‐face and digital components)?

- What steps would be best suited to face‐to face, and what steps would be better digitally?
- What are the tipping points for in‐person versus virtual?
- What elements need to be in person? What elements can be done through live video conference with your health care team? What elements can be purely digital and not involve live communication with your healthcare team?
- What if digital care could potentially improve the quality of your in‐person visits? Some examples: Completing administrative information prior to the visit with the doctor so that the time spent with the doctor can be used more wisely; receiving basic results online ahead of the in‐person meeting so there is time to research and prepare questions *(only offer this option after probing in earlier question about receiving results completely virtually)*
- Features/Design

### 2 Virtual care ± genetic testing (Key themes)

If you were asked to interact with a digital genetics tool…

Legitimacy/trust of data/platform:

- What would make you trust this platform?
- What makes you think a platform is credible?
- What are your biggest privacy concerns?
- What privacy and security features need to be part of the platform for you to trust it?
- How do you feel about a tool that uses information you input to decide what information to show you next? Provide targeted information.
  ⚬ Any concerns?
  ⚬ Who has access to it?
  ⚬ Security?

How to digitalize and humanize the platform:

- What is required to give the platform a human touch?
- How can we humanize theplatform?
- Language, introduction/avatar
- Is this a deal breaker?

Chatbot:

- Explain what a chatbot is (i.e., you ask a question and the chatbot (computer) answers)
- What would make you comfortable engaging with a chatbot?
- What types of things do you envision asking a chatbot regarding genetic services? *(Probes: educational, decisional, and emotional support)*
- In what ways would talking with a chatbot be better than talking with a real person? In what ways would it be more difficult? What would help you develop a relationship with a chatbot?
- Who is the chatbot in your mind? Is it an avatar of a doctor, GC, woman/male? What is their “persona”? Or are they ambiguous?
